# Supplementary material for: Influenza Vaccination Coverage Rates and Determinants in Greek Children until the Age of Ten (2008–2019), the Rhea Mother–Child Cohort
Source: Vaccines (Basel). 2023 Jul 14;11(7):1241. doi: 10.3390/vaccines11071241 (PMC10384674; doi:10.3390/vaccines11071241)
Supplement: Supplementary file 1 [file vaccines-11-01241-s001.zip › vaccines-2491389-supplementary.pdf]

Supplementary material

This supplementary material is hosted by Vaccines as supporting information alongside the article "Influenza vaccination coverage rates and determinants in Greek children until the age of ten (2008-2019); the Rhea mother-child cohort", on behalf of the authors, who remain responsible for the accuracy and appropriateness of the content. The same standards for ethics, copyright, attributions and permissions as for the article apply.

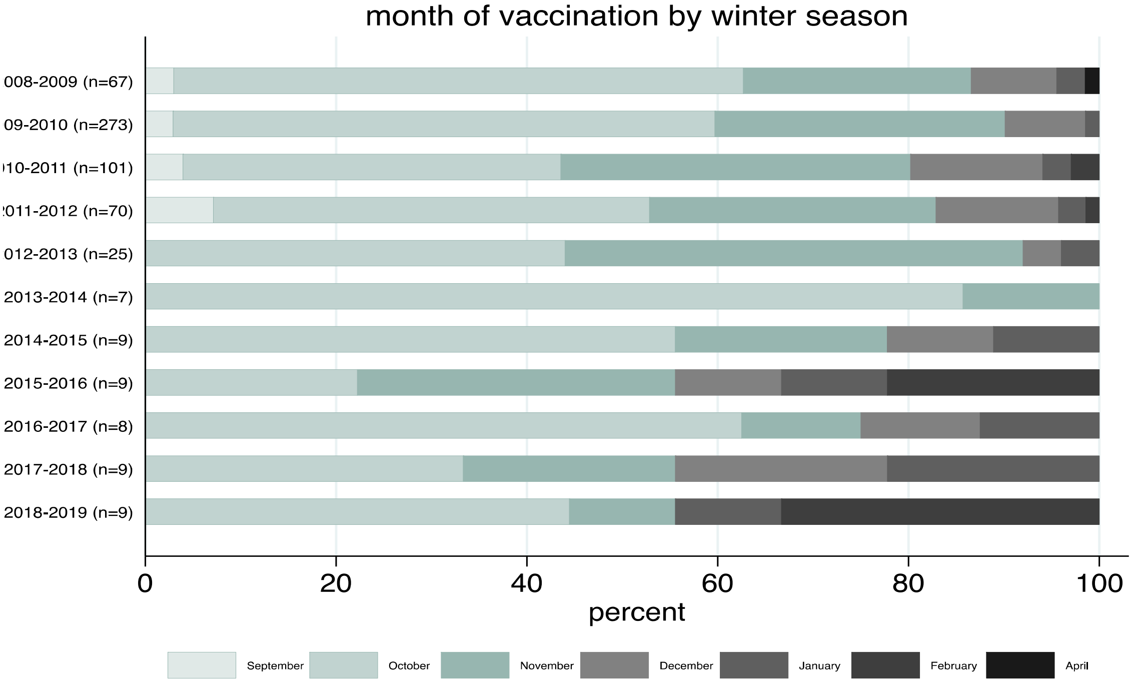

Supplementary Figure S1. Distribution of vaccinations by month of vaccination in each winter season. The season 2019-2020 was excluded from this analysis and graph, because only a few children were evaluated at this season.

**Supplementary table S1.** Frequency of trades among all vaccine doses administered		
**trade**	**n**	**%**
vaxigrip	733	80.1
fluarix	26	2.8
focetria	11	1.2
influvac	2	0,2
unknown	143	15.7
Total	915	100

Supplementary Table S2. Determinants of ever vaccination by age four based on univariate regression models.

	all	Ever vaccinated until age four		
Characteristics	n (%)	n (%)	OR	95% CI
**maternal age**				
35-45	97 (11,8)	30 (9,8)	0.74	[0.43,1.26]
25-35	558 (68,1)	214 (69,9)	1.02	[0.71,1.47]
17-25	164 (20,0)	62 (20,3)		REF
**Maternal education**				
high	261 (32,5)	104 (35,0)	1.55	[0.99,2.44]
medium	414 (51,6)	155 (52,2)	1.40	[0.91,2.15]
low	127 (15,8)	38 (12,8)		REF
**Maternal origin**				
Greek	772 (94,1)	288 (95,0)	1.31	[0.70,2.45]
non-Greek	48 (5,9)	15 (5,0)		REF
**Gender**				
girl	405 (49,0)	147 (47,9)	0.93	[0.70,1.24]
boy	422 (51,0)	160 (52,1)		REF
**preterm birth**				
yes	95 (11,8)	40 (13,6)	1.29	[0.84,2.00]
no	709 (88,2)	255 (86,4)		REF
**delivery type**				
caesaric	400 (49,5)	142 (47,8)	0.90	[0.67,1.20]
vaginal	408 (50,5)	155 (52,2)		REF
**breastfeeding ever**				
yes	672 (86,5)	244 (85,0)	0.82	[0.54,1.25]
no	105 (13,5)	43 (15,0)		REF
**older siblings**				
yes	445 (55,4)	150 (50,8)	0.75*	[0.56,1.00]
no	358 (44,6)	145 (49,2)		REF
**child's health insurance**				
"IKA"	346 (42.2%)	105 (34.4%)	0.53***	[0.37, 0.74]
"OGA"	136 (16.6%)	59 (19.3%)	0.93	[0.61, 1.42]
"OPAD/OAEE"	232 (28.3%)	105 (34.4%)		REF
other	93 (11.3%)	31 (10.2%)	0.60*	[0.37, 1.00]
none	13 (1.6%)	5 (1.6%)	0.76	[0.24, 2.38]
**passive smoking 4 years**				
yes	368 (46,2)	121 (41,0)	0.72*	[0.53,0.96]
no	428 (53,8)	174 (59,0)		REF
**residency 4 years**				
urban	588 (71,1)	210 (68,4)	0.81	[0.60,1.11]
rural	239 (28,9)	97 (31,6)		REF
**asthma diagnosis 4 years**				
yes	44 (5,4)	19 (6,3)	1.30	[0.71,2.41]
no	771 (94,6)	284 (93,7)		REF
**obese 4 years**				

yes	60 (7,3)	23 (7,5)	1.06	[0.62,1.82]
no	764 (92,7)	282 (92,5)		REF

Supplementary Table S3. Determinants of ever vaccination by age four based on stepwise regression models among children without asthma and/or obesity (n=501 with complete data)

	Univariate		Multivariate	
**Characteristics**	OR	95% CI	OR	95% CI
**early daycare attendance**				
yes	1.46*	[1.02, 2.09]	1.51	[0.97, 2.35]
no		REF		REF
**younger siblings**				
yes	1.62**	[1.18, 2.21]	1.75**	[1.19, 2.59]
no		REF		REF
**passive smoking 1 year**				
yes	0.70*	[0.51, 0.97]	0.70	[0.48, 1.02]
no		REF		REF
**respiratory infections the first year**				
2-4 infections	1.75**	[1.16, 2.64]	2.08**	[1.32, 3.29]
0-1 infections		REF		REF
**respiratory infections the fourth year**				
2-4 infections	1.49*	[1.05, 2.10]	1.44	[0.94 ,2.21]
0-1 infections		REF		REF
